# Circulating levels of adipose tissue-derived inflammatory factors in elderly diabetes patients with carotid atherosclerosis: a retrospective study

**DOI:** 10.1186/s12933-018-0723-y

**Published:** 2018-05-30

**Authors:** Wei Yang, Yun Li, Jie-Yu Wang, Rui Han, Li Wang

**Affiliations:** 1 Department of Geriatric Medicine, Capital Medical University, Xuanwu Hospital, No. 45 Chang Chun Street, Beijing, 100053 China; 2 Department of Endocrine, Capital Medical University, Xuanwu Hospital, Beijing, 100053 China

**Keywords:** Vaspin, Resistin, Visfatin, Diabetes mellitus, Atherosclerosis

## Abstract

**Background:**

Inflammation has been recognized as a key feature of both type 2 diabetes mellitus (T2DM) and atherosclerosis. However, the relationships between circulating levels of novel adipose tissue-derived inflammatory factors, including resistin, vaspin, and visfatin, and the severity of atherosclerosis have not been determined. Moreover, the associations between these inflammatory factors and obesity and insulin resistance in elderly patients remain to be clarified.

**Methods:**

A cross-sectional study of 256 elderly patients with T2DM admitted in our center was performed. Baseline circulating levels of resistin, vaspin and visfatin were measured with enzyme-linked immunosorbent assays. Ultrasonic evaluations of the carotid arteries of the patients were performed to reflect the severity of systemic atherosclerosis. Patients were classified as having mild, moderate, or severe atherosclerosis according to the results of carotid ultrasonic examination. Circulating levels of the inflammatory factors listed above also were correlated with body mass index (BMI) and homeostasis model assessment of insulin resistance (HOMA-IR).

**Results:**

With more severe carotid atherosclerosis, circulating levels of resistin (mild: 2.01 ± 0.23; moderate: 2.89 ± 1.01; severe: 3.12 ± 1.12; p < 0.05) and visfatin (mild: 11.63 ± 7.48; moderate: 15.24 ± 2.19; severe: 17.54 ± 2.98; p < 0.05) gradually increased, while level of vaspin decreased (mild: 317 ± 23.12; moderate: 269 ± 32.12; severe: 229 ± 14.24; p < 0.05). Subsequent results of Pearson coefficient analyses indicated that all of the tested adipose tissue-derived inflammatory factors were positively correlated with the BMI and HOMA-IR of the patients (all p < 0.05), even after adjustment for hs-CRP.

**Conclusions:**

The adipose tissue-derived inflammatory factors resistin, vaspin and visfatin may be involved in the pathogenesis of atherosclerosis in elderly T2DM patients.

## Background

Atherosclerosis has been recognized as a fundamental pathologic change in many cardiovascular diseases [1], and thus, represents an important threat to the health of the global population. Indeed, the primary pathophysiological feature of both coronary heart disease (CHD) and stroke is atherosclerosis, and these diseases have become major contributors to morbidity and mortality worldwide [2, 3], particularly among the elderly population [4]. Conventionally, many risk factors have been identified for the pathogenesis of atherosclerosis, including male gender, family history of early incidence of cardiovascular diseases, smoking, hypertension, dyslipidemia, obesity, and diabetes mellitus (DM) [5]. Atherosclerosis is now considered an inflammatory disease, because overactivated inflammation has been demonstrated to be a common pathway that mediates the pathogenesis of atherosclerosis [6, 7]. Therefore, identification of key inflammatory mediators involved in the initiation and progression of atherosclerosis may be of important clinical significance for the prevention and treatment of atherosclerosis-related cardiovascular diseases [8].

Type 2 DM (T2DM) is characterized by insulin resistance and has been confirmed to be a strong risk factor for the pathogenesis of atherosclerosis [9]. More than 400 million people globally are estimated to DM, and data from a recent epidemiological study suggest that the age-standardized prevalence of total DM is nearly 10% and the prevalence in people over 60 years old exceeds 20% [10]. Development of vascular disease has become a major cause of morbidity and mortality in patients with T2DM. Indeed, a previous collaborative meta-analysis of 102 prospective studies showed that compared with controls without T2DM, patients with T2DM have a 73% increased risk for total vascular diseases, including a 100% increased risk for CHD, a 127% increased risk for ischemic stroke, and a 54% increased risk for hemorrhagic stroke [11]. However, the potential mechanisms underlying the association between T2DM and atherosclerosis have not been fully determined, and the activated inflammatory response has been considered an important common pathophysiological feature of both diseases [12, 13]. Indeed, classical inflammatory factors, such as the high-sensitivity C-reactive protein (hs-CRP) and tumor necrosis factor alpha are involved in the pathogenesis of both T2DM and CHD [14, 15]. Moreover, adiponectin, an inflammatory peptide secreted by adipocytes, and neutrophil gelatinase-associated lipocalin (NGAL), an acute phase protein released by neutrophils, are involved in the interrelationship between T2DM and CHD [16, 17]. These findings raised the hypothesis that anti-inflammatory treatment may be effective for the prevention of both T2DM and CHD [18]. Importantly, the recently published Canakinumab Anti-inflammatory Thrombosis Outcomes Study (CANTOS) showed that anti-inflammatory treatment with Canakinumab targeting the interleukin-1β innate immunity pathway reduced the risk of recurrent cardiovascular events in patients with previous myocardial infarction [19]. However, the incidence of T2DM was not significantly affected [20], indicating the importance of identifying novel inflammatory factors that mediate the association between T2DM and CHD. Recent evidence suggests that in addition to storing energy, adipose tissue may secrete several inflammatory factors, such as resistin, vaspin and visfatin, which may be important mediators of obesity, atherosclerosis, and DM [21, 22]. Resistin was initially discovered as an adipocyte-secreted hormone mediating obesity and insulin resistance in animal studies, and a recently published study in humans suggests its potential role in the pathogenesis of atherosclerosis [23]. Vaspin, a visceral adipose tissue-derived serine protease inhibitor that is upregulated in animal models of obesity and insulin resistance, has also been hypothesized to participate in the development of atherosclerosis [24]. Moreover, visfatin, also known as the enzyme nicotinamide phosphoribosyltransferase (Nampt), which was initially identified as a molecule with insulin-like properties in 2005, also has been suggested to be involved in the pathogenesis of atherosclerosis primarily by mediating the inflammatory response [25, 26]. However, the circulating levels of these inflammatory factors in T2DM patients according to the severity of atherosclerosis have not been determined. More importantly, the associations between these adipose-derived inflammatory factors and the severity of atherosclerosis in elderly patients with T2DM have not been reported. The aim of the current retrospective cohort study was to compare the levels of resistin, vaspin, and visfatin in T2DM patients according to the severity of carotid atherosclerosis and to explore the potential relationships of these factors and the conventional risk factors for T2DM and atherosclerosis, such as body mass index (BMI) and indicators of insulin resistance.

## Methods

This retrospective cross-sectional study included 256 elderly patients (> 60 years) with T2DM who were admitted to the Department of Geriatrics or Department of Endocrinology of Xuanwu Hospital affiliated to the Capital Medical University between July 2013 and July 2017. Written informed consent was obtained from each included patient before enrollment. The study protocol was approved by the Ethics Committee of Xuanwu Hospital affiliated to the Capital Medical University before the performance of the study.

### Inclusion and exclusion criteria of the patients

Patients were included if they were > 60 years and diagnosed with T2DM according to the criteria of 2013 Chinese Guidelines for the Management of Diabetes [27]. Specifically, patients were diagnosed with T2DM if they met either of the following criteria: (1) presented with symptoms of hyperglycemia (dry mouth, polydipsia, polyuria, and weight loss) and random plasma glucose ≥ 11.1 mmol/L; or (2) fasting plasma glucose (FPG) ≥ 7.0 mmol/L; or (3) 2-h postprandial plasma glucose (2 h-PPG) ≥ 11.1 mmol/L on standardized oral glucose tolerance test (OGTT). Patients were excluded if they had any of the following clinical conditions: type 1 DM; comorbidities of acute complications of T2DM; other acute clinical conditions or severe diseases such as severe hepatic or renal dysfunction, severe infection, sepsis, or malignancies; other endocrine or autoimmune diseases that may affect the systemic levels of adipose tissue-derived inflammatory factors; or taking hormonal preparations or immune inhibitors with the potential to affect the systemic levels of inflammatory factors, such as glucocorticoid, cyclosporine A, or tacrolimus etc. at enrollment.

### Definitions of clinical parameters

Clinical parameters, including demographic data (age, gender, body weight, and height), BMI, duration of T2DM, waist to hip circumference ratio (WHR), systolic blood pressure (SBP), diastolic blood pressure (DBP), and blood biochemical parameters of lipids and glucose metabolism, were obtained from individual patients at admission. Briefly, BMI was calculated by dividing the weight (kg) by the square of the height (m^2^). For each patient, we measured waist and hip circumferences twice with an inextensible tape. The patients were instructed to stand erect with arms relaxed at both sides and feet close together. To measure the waist circumference, the tape was placed just above the uppermost lateral border of the right iliac crest in a horizontal plane around the abdomen, and the measurement was performed at the end of a normal expiration. To measure the hip circumference, the tape was placed at the level of the greater trochanter, which indicated the position of the middle part of the hip in a horizontal plane. During the measurements, the tape was placed at a horizontal level parallel to the floor. The measurements were recorded in centimeters (cm) to the nearest 0.1 cm. Information regarding the prescription of medications for the primary prevention of cardiovascular diseases and treatment of T2DM was also recorded, such as the use of aspirin, statins, probucol, metformin, acarbose, sulfonylureas (SUs), thiazolidone (TZDs), and insulin.

### Measurement of blood biochemical parameters

We obtained blood samples from all included patients between 6 a.m. and 10 a.m. at the admission of the patients after fasting for more than 12 h. The blood samples were centrifuged immediately for further analyses of the blood biochemical parameters of lipids and glucose metabolism, and the serum samples were stored at − 80 °C for measurements of adipose tissue-derived inflammatory factors, including resistin, vaspin, and visfatin. A standardized OGTT with a 75-g oral glucose load was performed for each patient to measure the 2 h-PPG. Briefly, FPG and 2 h-PPG were measured with a glucose oxidase procedure. Glycosylated hemoglobin (HbA1c) was measured with a Cobas Integra 800 automated biochemistry analyzer (Roche, Basel, Switzerland) according to the manufacturer’s instruction. We used the radioimmunoassay analysis method (Ray Bio, Norcross, GA) to measure the fasting serum insulin (FSI) concentration, and an enzyme-linked immunosorbent assay (ELISA) was applied for the measurement of hs-CRP. Parameters for the indexes of lipid metabolism, including total cholesterol (TC), triglyceride (TG), low-density lipoprotein (LDL-C), and high-density lipoprotein (HLD-C), were measured with a Hitachi 7600 automatic biochemical analyzer following the instructions of the manufacturer. To represent the extent of insulin resistance of each included patient, we calculated the parameter of insulin resistance index (homeostasis model assessment of insulin resistance, HOMA-IR) according to the following equation: HOMA-IR = FPG (mmol/L) × FINS (μU/L)/22.5.

### Determination of circulating resistin, vaspin and visfatin levels

A partial blood sample for each patient was used for measurements of circulating vaspin, resistin, and visfatin levels. Briefly, the serum of the blood sample was obtained after centrifugation at 3500 rpm for 5 min within 2 h after blood collection. The serum levels of resistin, vaspin, and visfatin were measured with commercially available ELISA kits in an automatic multifunctional enzymatic standard instrument (Thermo MK3, USA).

### Ultrasonic evaluation of the carotid arteries

All of the included patients underwent ultrasonic evaluation of the carotid arteries to determine the extent of systemic atherosclerosis. The ultrasonic evaluation of the carotid arteries was performed by an experienced physician for each included patient on the first day of their admission. The patients were classified according to the severity of atherosclerosis as reflected by the findings of the ultrasonic evaluation of the carotid arteries: (1) mild group: thickening of the intima-media or plaque formation in the carotid artery; (2) moderate group: arterial stenosis of < 50% in the carotid artery of either side; and (3) severe group: arterial occlusion or arterial stenosis of ≥ 50% in the carotid artery of either side.

### Statistical analyses

We used SPSS for Windows Software, Version 18.0 (SPSS Inc., Chicago, IL, USA) for statistical analyses. Data are presented as mean ± standard deviation (SD) if they were normally distributed. For data that were not normally distributed, logarithmical transformation was performed to achieve normal distribution. We applied single factor analysis of variance to compare the quantitative data among multiple groups. T test was applied to compare the quantitative data between two groups. Fisher’s exact test was applied for the analysis of categorical variables. Single factor correlation between two independent variables was analyzed with Pearson coefficient analysis. Moreover, correlations between the three adipose tissue-derived inflammatory factors and metabolic parameters were also analyzed after adjustment for hs-CPR levels. A *p* value less than 0.05 was considered statistically significant.

## Results

### Characteristics of the patients according to the severity of carotid atherosclerosis

Overall, our study included 256 elderly patients with T2DM who were admitted in our hospital, of which 87, 105, and 64 patients were allocated to the mild, moderate, and severe atherosclerosis groups according to the findings of ultrasonic examination of the carotid arteries. The demographic characteristics and the clinical parameters of the included T2DM patients according to the severity of atherosclerosis are presented in Tables 1 and 2. No significant differences were detected regarding age, gender, duration of BMI, or DBP among patients allocated to the above three groups (all p > 0.05). However, compared with those with mild to moderate atherosclerosis, elderly T2DM patients with severe atherosclerosis were more likely to have a higher BMI, WHR, SBP, and TG, as well as lower HDL, indicating that severe atherosclerosis is more likely to be complicated by conventional risk factors of atherosclerosis, such as obesity, hypertension, and dyslipidemia. Moreover, higher levels of FINS and HOMA-IR were noticed in patients with severe atherosclerosis, although HbA1c did not differ significantly among the patients of the three groups. These results suggest that elderly T2DM patients with severe atherosclerosis have more significant insulin resistance, although the statuses of management of T2DM across the groups did not significantly differ. More importantly, we found that circulating levels of the adipose tissue-derived inflammatory factors resistin and visfatin were significantly higher in patients with severe atherosclerosis; however, the level of vaspin was significantly lower in these patients. These results, together with the finding of a significantly increased level of hs-CRP, a classical inflammatory factor involved in atherosclerosis, demonstrated that overactivated systemic inflammation, including changes in adipose tissue-derived inflammatory factors, may be involved in the pathogenesis of microvascular complications in elderly patients with T2DM.

**Table 1:** Baseline characteristics of patients according to the severity of carotid atherosclerosis

	Mild group	Moderate group	Severe group	F value/χ^2^ p
	(n = 87)	(n = 105)	(n = 64)	
Gender (M/F)	55/32	64/44	40/24	0.653 > 0.05
Age (years)	75.52 ± 3.32	76.21 ± 7.21	76.01 ± 5.21	0.487 > 0.05
DM duration (years)	23.89 ± 2.32	23.28 ± 1.26	24.79 ± 3.43	0.563 > 0.05
BMI (kg/m^2^)	25.13 ± 2.54	26.40 ± 5.24	28.24 ± 5.14*	8.324 < 0.05
WHR	0.83 ± 0.13	0.86 ± 0.24	0.98 ± 0.65*^△^	11.324 < 0.01
SBP (mmHg)	135 ± 6	141 ± 13	148 ± 12*	4.213 < 0.05
DBP (mmHg)	69 ± 4	67 ± 7	72 ± 9	0.672 > 0.05
Medications, n (%)				
Aspirin	26 (29.8%)	54 (51.4%)	48 (75%)	*6.324 < 0.05*
Statins	34 (39%)	55 (52%)	50 (78.1%)	7.324 < 0.01
Probucol	10 (11.4%)	25 (23.8%)	40 (62.5%)	5.234 < 0.05
Metformin	35 (40.2%)	74 (70.4%)	55 (85.9%)	5.341 < 0.05
Acarbose	55 (63.2%)	76 (72.3%)	55 (85.9%)	0.985 > 0.05
SUs	15 (17.2%)	27 (25.7%)	16 (17.8%)	0.765 > 0.05
TZDs	35 (40.2%)	57 (54.2%)	48 (75%)	2.543 < 0.05
Insulin	22 (25.2%)	46 (43.8%)	45 (70.3%)	4.324 < 0.05

Italic values indicates that, the proportions of patients that received aspirin and probucol were significantly larger in patients with severe atherosclerosis as compared with those in patients with mild atherosclerosis; while the proportions of patients were significantly different among the three groups. As for the hypoglycemic, the proportions of patients that received metformin, thiazolidones and insulin injection were significantly larger as compared with those in patients with mild atherosclerosis

*DM* diabetes mellitus, *BMI* body mass index, *WHR* waist hip ratio, *SBP* systolic blood pressure, *DBP* diastolic blood pressure, *SUs* sulfonylureas, *TZDs* thiazolidone

* p < 0.05 compared with the mild group; ^Δ^ p < 0.05 compared with the moderate group

**Table 2:** Circulating levels of vaspin, resistin, and visfatin and other parameters related to metabolism and inflammation according to the severity of carotid atherosclerosis

	Mild group (n = 87)	Moderate group (n = 105)	Severe group (n = 64)	F value/χ^2^ p value
TG (mmol/L)	1.50 ± 0.69	1.64 ± 1.24	2.34 ± 1.41^Δ#^	7.745 < 0.05
TC (mmol/L)	4.76 ± 0.23	4.61 ± 0.54	4.75 ± 2.09	0.876 > 0.05
LDL-C (mmol/L)	2.62 ± 0.93	2.61 ± 0.75	2.59 ± 1.17	0.654 > 0.05
HDL-C (mmol/L)	1.79 ± 0.59	1.58 ± 0.45	1.37 ± 0.50^Δ#^	5.567 < 0.05
FPG (mmol/L)	7.11 ± 1.20	7.76 ± 3.16	8.76 ± 2.20	0.532 > 0.05
FINS (mIU/L)	21.73 ± 5.2	22.78 ± 11.72	26.89 ± 13.12^Δ^	4.543 < 0.05
HOMA-IR	7.41 ± 2.72	9.44 ± 21.18^Δ^	13.17 ± 6.62^Δ#^	7.324 < 0.05
HbA1C (%)	7.58 ± 0.41	8.07 ± 1.97	8.42 ± 2.17	0.432 > 0.05
Vaspin (pg/mL)	317 ± 23.12	269 ± 32.12	229 ± 14.24^Δ^	9.511 < 0.05
Resistin (ng/mL)	2.01 ± 0.23	2.89 ± 1.01	3.12 ± 1.12^Δ^	7.271 < 0.05
Visfatin (µg/mL)	11.63 ± 7.48	15.24 ± 2.19^Δ^	17.54 ± 2.98^Δ^	6.876 < 0.05
hs-CRP (mg/L)	2.78 ± 3.84	3.12 ± 4.30	5.02 ± 3.54^Δ#^	5.321 < 0.05

*TG* triglyceride, *TC* total cholesterol, *LDL-C* low-density lipoprotein cholesterol, *HLD-C* high-density lipoprotein cholesterol, *FPG* fasting plasma glucose, *FINS* fasting insulin, *HOMA-IR* homeostasis model assessment of insulin resistance, *HbA1C* glycosylated hemoglobin, *hs-CRP* high-sensitivity C-reactive protein

^Δ^p < 0.05 compared with the mild group; ^#^ p < 0.05 compared with the moderate group

### Correlations between circulating vaspin, resistin, and visfatin levels

To further clarify the relationships of the levels of the three adipose tissue-derived inflammatory factors in elderly patients with T2DM, Pearson coefficient analyses were performed. We found that levels of resistin and visfatin were significantly correlated in these patients (R linear = 4.356, p = 0.043), whereas neither of resistin nor visfatin levels were significantly correlated with the level of vaspin (both p > 0.05).

### Correlations of circulating vaspin, resistin, and visfatin levels with BMI and HOMA-IR

**Fig. 1:**
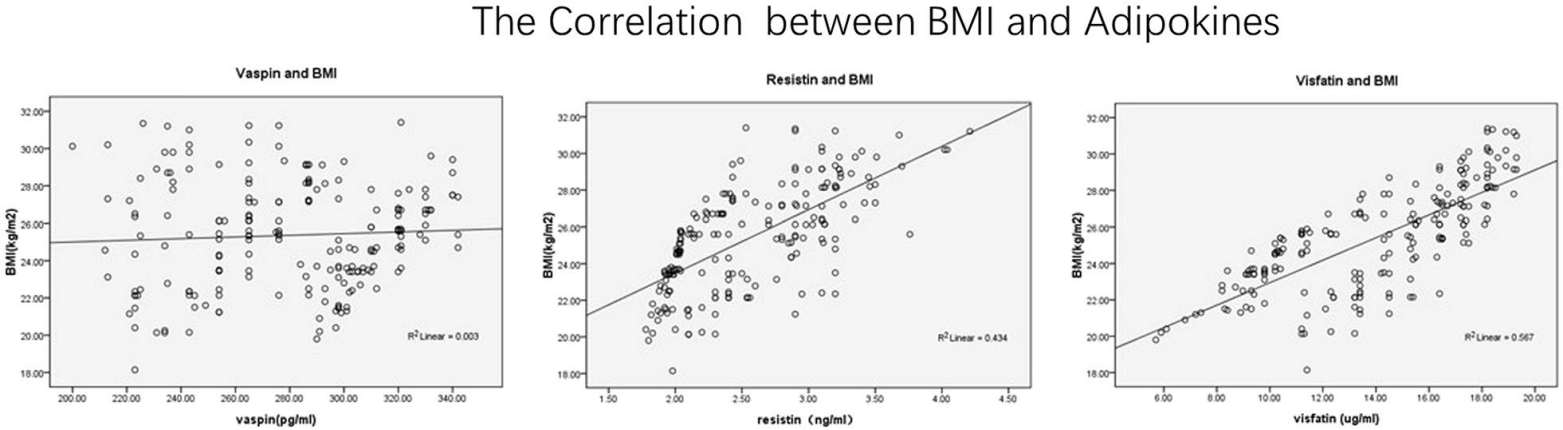
Correlations of circulating vaspin, resistin, and visfatin levels with BMI in elderly patients with T2DM

**Fig. 2:**
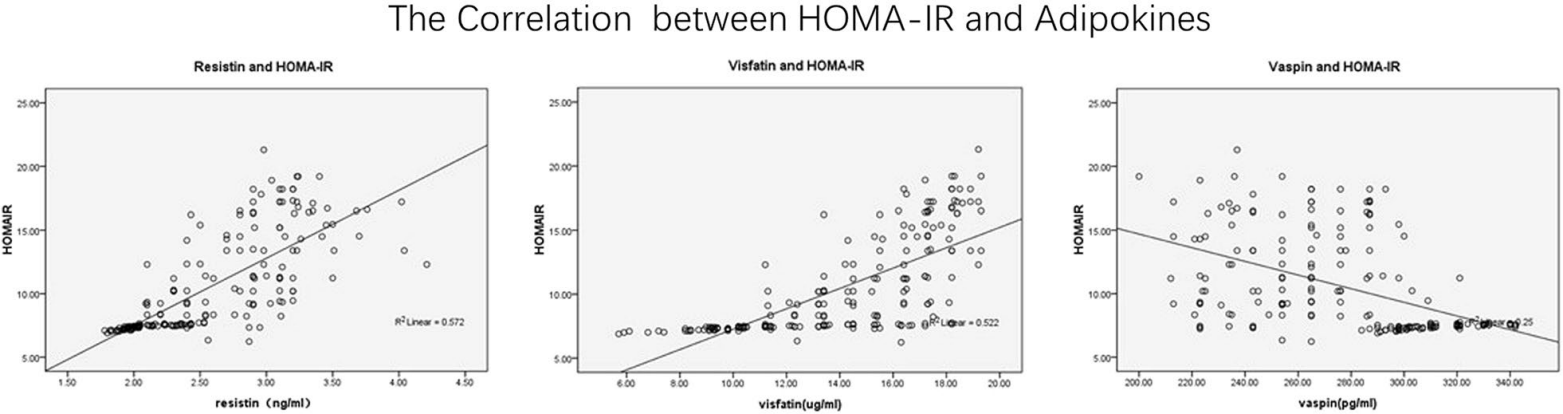
Correlations of circulating vaspin, resistin, and visfatin levels with HOMA-IR in elderly patients with T2DM

To further clarify the significance of changes in levels of adipose tissue-derived inflammatory factors in elderly patients with T2DM, Pearson coefficient analyses were performed to elucidate the association between adipose tissue-derived inflammatory factors and conventional risk factors for atherosclerosis. We found that fasting serum vaspin was positively correlated with gender and TG, but not with other variables including age, duration of T2DM, WHR, TC, LDL-C, FPG, HbA1c, or hs-CRP (Table 3). Similarly, fasting serum resistin was positively correlated with TG, but not with gender, age, duration of T2DM, WHR, TC, LDL-C, FPG, HbA1c, or hs-CRP (Table 3). Moreover, fasting serum visfatin was positively correlated with WHR, but not with gender, age, duration of T2DM, TG, TC, LDL-C, FPG, HbA1c, or hs-CRP (Table 3). Importantly, all of the above adipose tissue-derived inflammatory factors showed positive correlations with BMI and HOMA-IR (Figs. 1, 2), suggesting that activated inflammation in elderly patients with T2DM may be associated with obesity and insulin resistance in these patients. Subsequent analyses after adjustment for hs-CRP showed similar results (Table 4), suggesting the correlations of the above adipose tissue-derived inflammatory factors with BMI and HOMA-IR were independent of the systematic inflammation index.

**Table 3:** Correlations between circulating vaspin, resistin, and visfatin levels and the other parameters related to metabolism and inflammation in elderly patients with T2DM

	Statistic	Gender	TG	WHR	BMI	HOMA-IR
Vaspin	R value	0.855	0.857		0.88	0.776
	p value	0.014	0.023		0.029	0.032
Resistin	R value		0.963		0.812	0.724
	p value		0.024		0.041	0.047
Visfatin	R value			0.814	0.898	0.821
	p value			0.041	0.037	0.039

*TG* triglyceride, *HOMA*-*IR* homeostasis model assessment of insulin resistance, *BMI* body mass index, *WHR* waist hip ratio

**Table 4:** Correlations between circulating vaspin, resistin, and visfatin levels and other parameters related to metabolism and inflammation in elderly patients with T2DM after adjustment for hs-CRP

	Statistic	TG	WHR	BMI	HOMA-IR
Vaspin	R value	0.731		0.786	0.721
	p value	0.033		0.039	0.036
Resistin	R value	0.863		0.712	0.714
	p value	0.029		0.043	0.048
Visfatin	R value		0.772	0.828	0.842
	p value		0.045	0.039	0.032

*TG* triglyceride, *HOMA*-*IR* homeostasis model assessment of insulin resistance, *BMI* body mass index, *WHR* waist-to-hip ratio

## Discussion

In this cross-sectional study of elderly patients with T2DM, we found that circulating levels of novel adipose tissue-derived inflammatory factors showed a trend of significant change according to the severity of systemic atherosclerosis. Specifically, with the worsening of carotid atherosclerosis, circulating levels of resistin and visfatin gradually increased, while the level of vaspin decreased. Subsequent results of Pearson coefficient analyses indicated that levels of resistin and visfatin were positively correlated, and all the above adipose tissue-derived inflammatory factors were positively correlated with the BMI and HOMA-IR of the patients, even after adjustment for hs-CRP. These results suggest that resistin and visfatin, both as the proinflammatory factors, may exert a synergetic effect during the pathogenesis of atherosclerosis in these patients, and the circulating levels of resistin, vaspin, and visfatin may parallel the severity of systemic atherosclerosis, despite their roles as indicators of the extent of insulin resistance. Based on above findings, we hypothesize that resistin, vaspin and visfatin may be involved in the pathogenesis of atherosclerosis in elderly patients with T2DM.

Resistin was initially identified in white adipocytes of mice [28], and the serum level of resistin was found to be more remarkable in animal models of obesity and insulin resistance [29]. Subsequent experimental studies showed that resistin may be a mediator of insulin resistance, likely via a decrease in the phosphorylation of 5′ adenosine monophosphate-activated protein kinase (AMPK) in the liver [30]. This is consistent with our results, which showed a positive correlation between the circulating resistin level and the indicator of insulin resistance HOMA-IR in elderly patients with T2DM. Moreover, some evidence from experimental studies also suggests that resistin may accelerate the pathogenesis of atherosclerosis by promoting endothelial dysfunction, vascular smooth muscle cell proliferation, arterial inflammation, and the formation of foam cells [23]. Indeed, accumulating evidence from human epidemiological studies indicates that an increased circulating level of resistin may be related to increased risks of many cardiovascular diseases, including CHD and stroke [31]. A recently published prospective cohort study showed that higher resistin is a significant predictor of cardiovascular diseases independent of conventional risk factors in individuals over 70 years [32]. However, the predictive efficacy of resistin for cardiovascular risk was significantly attenuated by adjustment for inflammation [32]. We extended these findings by showing that the circulating resistin level increased in parallel with the severity of systemic atherosclerosis in elderly patients with T2DM, indicating that resistin may be an important contributor to vascular complication in these patients. In addition, visfatin/Nampt was initially identified as a substance with insulin-like properties in mice in 2005 [33]. Subsequent studies revealed that an important role of visfatin/Nampt is the regulation of the inflammatory response, likely via induction of other inflammatory factors, including interleukin-1, tumor necrosis factor alpha, interleukin-6, etc. [34]. The pro-inflammation efficacy of visfatin/Nampt has been considered to be the major mechanism underlying the induction of insulin resistance by visfatin. This was further confirmed by our findings, which showed a positive correlation between circulating visfatin and HOMA-IR. Interestingly, recently published studies in patients with T2DM showed that circulating visfatin is correlated with an increased intima-media thickness (IMT) of carotid arteries [35]. These findings were extended by our study, which showed that the circulating visfatin level increased gradually with the increasing severity of carotid atherosclerosis in elderly patients with T2DM. Moreover, a recent study in high-risk patients with ST segment elevated myocardial infarction suggested that the circulating level of visfatin in these patients can independently predict mortality risk [36]. The results of our study showed that circulating resistin and visfatin were positively associated with the severity of atherosclerosis in elderly diabetes patients. Interestingly, some recent prospective cohort studies also indicated that higher resistin and visfatin levels at baseline are associated with an increased risk of major cardiovascular adverse events (MACEs). In a prospective cohort study of 150 patients with diabetic nephropathy, increased resistin and visfatin levels at baseline were found to be independent predictors of cardiovascular mortality [37]. Moreover, the prospective association between baseline circulating resistin and the risk of MACEs has also been indicated in recent large cohort studies and a meta-analysis, particularly in T2DM patients [38, 39]. In summary, the inflammation-inducing efficacy of resistin and visfatin may be important for the development of atherosclerosis in patients with T2DM.

As a visceral adipose tissue-derived serine protease inhibitor, vaspin is also confirmed to be upregulated in animal models of obesity and insulin resistance [24]. Although the potential mechanisms underlying the effect of vaspin on insulin resistance remain to be determined, it has been suggested that the circulating level of vaspin correlates with the extent of insulin resistance in certain populations, such as overweight female patients with polycystic ovary syndrome [40]. However, no correlation between the circulating level of vaspin and HOMA-IR was observed in 108 subjects with normal glucose tolerance [41]. Our results were consistent with the previous study in that the circulating level of vaspin was correlated with HOMA-IR in elderly patients with T2DM. The discrepancy of the other results may be explained by the different study population included. However, whether other potential factors exist that confound the association between the level of vaspin and extent of insulin resistance deserves further investigation. Moreover, we found that the circulating level of vaspin decreased significantly in elderly T2DM patients increasing severity of atherosclerosis. This is inconsistent with the majority of previous findings, which showed that the circulating level of vaspin was positively associated with the severity of coronary stenosis in females with metabolic syndrome [42] and positively associated with the severity of carotid atherosclerosis in patients who underwent carotid endarterectomy [43]. Also, vaspin was shown to correlate with CAD in T2DM [44]. However, a previous cross-sectional study of T2DM patients showed an inverse association between serum vaspin and the presence of carotid plaque, which is similar to our findings [45]. Again, differences in the populations studied may contribute to the heterogeneity of the results, although other factors that may confound the association between the circulating level of vaspin and severity of atherosclerosis deserve investigation in future studies.

The strengths of our study include enrollment of elderly patients with T2DM who had rarely participated in previous similar studies. Moreover, we analyzed the changes in circulating levels of three novel adipose tissue-derived inflammatory factors to comprehensively evaluate their association with the severity of atherosclerosis in these elderly patients. Despite the above strengths, our study also has limitations that should be considered when interpreting the results. First, as a cross-sectional study, we could not examine the causative relationships of resistin, vaspin and visfatin with atherosclerosis or insulin resistance. Moreover, we did not perform multivariate adjusted analyses for the potential associations of resistin, vaspin and visfatin with atherosclerosis or insulin resistance. Therefore, we cannot not exclude the possibility that confounding factors, such as differences in lifestyle or medication used in the included patients, may affect the associations. In addition, the sample size of the study was relatively small. Due to the small number of patients included in the groups with differing severity of atherosclerosis, the study may be statistically underpowered to evaluate the correlations in the three different groups according to the severity of atherosclerosis. Therefore, the correlations of the three adipose tissue-derived inflammatory factors with the parameters of obesity, insulin resistance, etc. were not evaluated. Finally, as a single-center study, the generalizing of the conclusion to patients from other centers should be done cautiously.

## Conclusions

In conclusion, we found that circulating levels of novel adipose tissue-derived inflammatory factors, including resistin, vaspin and visfatin, may change according to the severity of atherosclerosis in elderly patients of T2DM, and these factors were correlated with the degree of insulin resistance. These results suggest that resistin, vaspin and visfatin may be involved in the pathogenesis of atherosclerosis in elderly patients with T2DM. The potential predictive efficacy and treatment significance of resistin, vaspin and visfatin for vascular complications in elderly T2DM patients warrant further investigation.

## Abbreviations

CHD: coronary heart disease
DM: diabetes mellitus
T2DM: type 2 diabetes mellitus
BMI: body mass index
HOMA-IR: homeostasis model assessment of insulin resistance
OGTT: oral glucose tolerance test
WHR: waist to hip circumference ratio
SBP: systolic blood pressure
DBP: diastolic blood pressure
HbA1c: glycosylated hemoglobin
FSI: fasting serum insulin
ELISA: enzyme-linked immunosorbent assay
hs-CRP: high-sensitivity C-reactive protein
TC: total cholesterol
TG: triglyceride
LDL-C: low-density lipoprotein
HLD-C: high-density lipoprotein
AMPK: adenosine monophosphate-activated protein kinase
